# Assessing the Capability of Large Language Models in Answering Pediatric Critical Care Board-Style Questions

**DOI:** 10.21203/rs.3.rs-7714101/v1

**Published:** 2025-11-03

**Authors:** Daniela Chanci, Ronald Moore, Henry P. Foote, Matthew A. Goldstein, Karan R. Kumar, Alexandre T. Rotta, Christoph P. Hornik, Marybeth Burriss-West, Makenzie Hamilton, Rishikesan Kamaleswaran

**Affiliations:** 1 Department of Biomedical Engineering, Duke University, 2080 Duke University Road, Durham, 27708, NC, USA.; 2 Department of Surgery, Duke University School of Medicine, 40 Duke Medicine Circle, 27710, Durham, NC, USA.; 3 Department of Pediatrics, Duke University School of Medicine, 40 Duke Medicine Circle, 27710, Durham, NC, USA.; 4 Division of Pediatric Critical Care Medicine, Department of Pediatrics, NYU Grossman School of Medicine, 550 First Avenue, 10016, New York, NY, USA.

**Keywords:** Large Language Models, Pediatric critical care, PICU, Multiple-choice questions, Clinical question-answering, Benchmark

## Abstract

**Background:**

The potential of Large Language Models (LLMs) in medicine is often linked to massive, resource-intensive models. However, their practical application in specialized fields like pediatric critical care requires exploring the capability of more efficient, locally-deployable open-source alternatives. In this study, we evaluated the accuracy and clinical reasoning of open-source LLMs of varying sizes, specifically assessing if smaller, efficient models can perform comparably to larger ones on pediatric critical care multiple-choice questions.

**Methods:**

A set of 100 pediatric critical care MCQs across six clinical domains, i.e. calculation, diagnosis, ethics, management, pharmacology, and physiology, was curated by two pediatric specialists to evaluate eight open-source LLMs, ranging from 2 to 70 billion parameters. The LLMs were assessed using the overall and category-specific accuracy and clinical reasoning quality score based on a 5-points Likert scale. Additionally, two pediatric critical care fellows completed the MCQs for comparison. Cochran’s Q test, McNemar’s test, the Friedman test, and Cohen’s kappa were used for the statistical analysis.

**Results:**

While the largest model (Llama-3.3-70B) achieved the highest accuracy (78%; 95% CI, 69%−86%), a key finding was the performance of the much smaller, 14.7-billion parameter Phi-4. This efficient model was strikingly comparable, with 75% accuracy (95% CI, 65%−83%) and a similar reasoning score (4.40 vs 4.49/5). Both models’ performance was on par with pediatric critical care fellows. The LLMs excelled in ethics but struggled with calculations. Inter-rater reliability was excellent for the clinical reasoning assessment (κ = 0.92).

**Conclusions:**

Our findings demonstrate that smaller, efficient LLMs can approach the performance of much larger models and pediatric critical care fellows for complex pediatric critical care reasoning. This suggests a viable pathway for developing secure, locally-deployable decision support tools without relying on massive, proprietary systems. At the same time, these models hold potential as complementary resources for trainee education in pediatric critical care. However, their identified weaknesses, especially in calculations, underscore that rigorous, domain-specific validation is an essential prerequisite to ensure safe use in both clinical and educational contexts.

**Trial registration:**

Not applicable.

## INTRODUCTION

Large language models (LLMs) have gained immense popularity due to their reasoning and problem-solving capabilities across a wide range of complex tasks [1]. In healthcare, numerous studies have evaluated the performance of LLMs for clinical documentation, notes summarization, medical question-answering, decision support based on electronic health record (EHR) data, and as complementary tools for medical education and training [2–4]. However, the majority of these efforts focused on adult populations, with limited assessment of LLMs capabilities in pediatrics. This gap is further amplified in pediatric critical care due to several challenges including the inherent physiological variability across age groups, differences in disease presentation, and the limited availability of high-quality datasets [5].

Prior work has focused on evaluating LLMs for specific pediatric tasks such as medication dosage calculation [6], differential diagnosis generation [7], chronic disease explanation [8], Emergency Severity Index (ESI) prediction [9], question-answering and education for patients and parents [10, 11], and general pediatric multiple-choice questions (MCQs) [12]. However, the performance of LLMs on pediatric critical care board-style questions, a highly specialized area that is likely not adequately represented in general-domain training data, is yet to be investigated. Furthermore, most existing studies evaluate proprietary LLMs such as GPT-4 [13], which raise privacy concerns for real-world decision-support tools involving sensitive patient information, or very large models like PaLM 2 [14], which pose significant computational challenges for deployment in clinical settings.

In this work, we evaluate the ability of open-source LLMs reason through pediatric critical care MCQs using multiple prompting strategies. Our contributions are as follows: (i) We have collaborated with clinicians to create a PICU MCQs dataset to evaluate LLMs. (ii) We comprehensively evaluated several state-of-the-art open-source LLMs for the selected MCQs. (iii) We conducted additional evaluation on the best-performing models to analyze their clinical reasoning capabilities on different pediatric critical care question categories and their performance compared to pediatric critical care fellows. Therefore, we not only assess LLMs viability for decision-support but also explore their potential role in medical education, where they are expected to provide trainees with accessible, interactive, privacy-preserving tools.

We use the terms *LLM* and *model* interchangeably throughout this paper.

## METHODS

### Dataset

Our study evaluated the clinical reasoning capabilities of LLMs in MCQ answering tasks focused on pediatric critical care. The study was conducted in accordance with the Helsinki Declaration, and ethical approval was obtained from the Institutional Review Board (IRB) of Duke University (Pro00115678) with a waiver of informed consent. We collaborated with two pediatric critical care specialists to develop an MCQ dataset to ensure clinical relevance. Each question was carefully selected from existing question banks to reflect real-world decision-making scenarios commonly encountered in the pediatric intensive care unit (PICU). The dataset consists of MCQs that cover core topics in pediatric critical care including mechanical ventilation strategies, sepsis management, congenital heart disease, and pediatric pharmacology.

Each MCQ follows a standardized format, consisting of three components: (i) a fundamental clinical knowledge question or a PICU realistic case-based scenario; (ii) four to five answer options; and (iii) a single correct response. The questions are categorized into six major domains: calculation, diagnosis, ethics, management, pharmacology, and physiology.

### Large Language Models

We evaluated eight open-source, general-purpose, instruction-tuned LLMs with potential for future adaptation and deployment in clinical settings. The models were *Gemma-2–2B* [15], *Gemma-2–9B* [15], *Gemma-3–27B* [16], *Llama-3.1–8B* [17], *Llama-3.2–3B* [17], *Llama-3.3-70B* [17], *Phi-3.5-mini* [18], and *Phi-4* (Table 1) [19]. We intentionally selected LLMs spanning a wide range of model sizes and training corpus sizes to assess how scale impacts pediatric critical care reasoning performance [20]. Implementation details are found in Supplemental Methods.

### Prompting Paradigms

**Figure 1.**
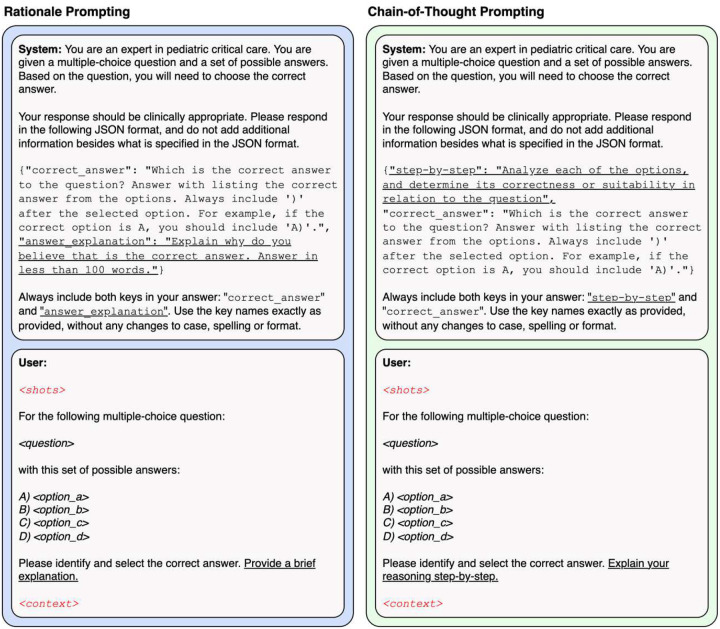
Prompt templates. Prompt template for the rationale and chain-of-thought prompting. The main differences between the two paradigms are underlined. For few-shot prompting, the selected shots are included in the field <shots> in red. Similarly, if using retrieval augmented generation, the retrieved context is included in the field <context> in red.

We designed and evaluated multiple prompting paradigms for the LLMs, including *zero-shot* and *few-shot* prompting [21]. as well as *rationale* and *chain-of-thought (CoT)* prompting [22]. These paradigms were also evaluated with Retrieval Augmented Generation (RAG) to enhance prompts by incorporating relevant external context (Figure 1) [23, 24]. However, no fine-tuning using domain-specific data was performed in this study. Prompt structure and design information is available in Supplemental Methods.

#### Zero-Shot Prompting

In this paradigm, the models are provided with system instructions and a single MCQ. They are expected to select the correct answer based only on the question and task description, without access to any few-shot examples [25].

#### Few-Shot Prompting

This approach leverages in-context learning by providing the models with 1 to 8 few-shot examples of pediatric critical care MCQs, formatted according to the template defined in the system content [21]. The examples generation process is included in Supplemental Methods. For consistency across model assessment, the same set of few-shot examples was used in all test MCQs.

#### Rationale Prompting

In this strategy, the LLMs are prompted to select the correct answer for a given MCQ and provide a brief explanation. Rationale prompting was applied in both zero-shot and few-shot paradigms, with or without RAG.

#### Chain-of-Thought Prompting

Here, the models are asked to reason through each answer option, and then select the correct one. This approach promotes reasoning, and as with rationale prompting, it was evaluated in zero-shot and few-shot settings, with and without and RAG [22].

### Retrieval Augmented Generation

Retrieval Augmented Generation (RAG) gained its popularity due to its ability to improve LLM performance without the need for fine-tuning [23]. RAG provides an LLM with knowledge relevant to the question as part of the input prompt. The process of constructing a RAG system involves three major steps, namely breaking down the collected data into chunks, vectorizing the data chunks, and storing the vectors in a vector database.

For our RAG system development, we used a reference pediatric critical care textbook [26]. We employed the recursive chunking method available in the Langchain Python library package to obtain chunk sizes of 200 characters. The 3 most relevant vectors using the gte-base-en-v1.5 multi-lingual embedding model and cosine similarity [27]. More details on the RAG pipeline and implementation are included in Supplemental Methods.

### Evaluation Metrics

We assessed the performance and clinical reasoning capabilities of the models in pediatric critical care MCQ using two metrics.

First, we calculated the accuracy, defined as the proportion of the questions for which the model selected the correct answer. This was computed using the answer choice included in the *correct_answer* field of the JSON-formatted model output. Accuracy was evaluated across the eight models and all prompting strategies included in this study.

Second, to quantitatively evaluate the clinical reasoning quality, a pediatric intensivist and a pediatric cardiologist independently rated the reasoning included in the *brief_explanation* or *step-by-step* fields using a 5-point Likert scale based on correctness and clinical relevance as follows:

1. Poor: The reasoning is totally incorrect, misleading, and/or irrelevant.
2. Fair: The reasoning contains significant clinical inaccuracies.
3. Acceptable: The reasoning is clinically accurate, but it is not related or relevant for the evaluated MCQ.
4. Good: The reasoning is clinically accurate and relevant, but it is missing important information that should have been included.
5. Excellent: The reasoning is clinically accurate, relevant, complete, and clear.

For this assessment, we randomly selected a stratified sample of 40 questions to ensure representation across different MCQs categories. Additionally, we focused on two models representing different scales: Phi-4 (small size), and Llama-3–70B (large size). We evaluated exclusively the zero-shot prompting strategy, with rationale and CoT prompting, without RAG, to examine the models’ inherent clinical reasoning capabilities using only their pretrained knowledge, beyond simply identifying the correct answer.

### Human Comparison

To provide a clinical performance benchmark, two pediatric critical care fellows completed the MCQs used for LLM evaluation independently. They answered the questions without time constraints or external resources, mimicking the zero-shot, rationale prompting strategy used for the LLMs.

### Statistical Analysis

The Cochran’s Q test was used to determine the performance differences across models and prompting strategies, followed by pairwise McNemar’s test with Benjamini-Hochberg adjustment when significant. Clinical reasoning scores were analyzed using the Friedman test. Agreement for the Likert ratings and the MCQs completions was quantified using Cohen’s kappa. Confidence intervals were calculated using the Wilson Score method.

## RESULTS

We created a dataset of 100 MCQs in pediatric critical care, each with 4 to 5 answer choices. The questions covered 6 different clinical categories: calculation, diagnosis, ethics, management, pharmacology, and physiology. Among these, calculation and ethics were the least represented, with 5 and 3 questions respectively, while management (32 questions) and physiology (31 questions) were the most prevalent categories (**Supplemental Table 1**). Out of the 100 questions, 8 were randomly selected to serve as few-shot examples, and the remaining 92 comprised the test set used to assess the LLMs.

All models were evaluated based on the accuracy of the selected answer choice for pediatric critical care MCQs. Table 2 presents the overall accuracy of the eight LLMs assessed under zero-shot learning, comparing both rationale and CoT prompting strategies, with and without RAG. Overall, larger models performed better. For instance, Llama-3.3-70B, the largest model assessed, consistently achieved the highest performance across all prompting strategies, with accuracies ranging from 74% (68 of 92; 95% CI, 64%−82%) to 79% (73 of 92; 95% CI, 70%−86%), marginally higher than Gemma-3–27B (*p* = 0.077). Notably, Phi-4 despite having substantially fewer parameters (14.7B), achieved an accuracy on par with Llama-3.3-70B (*p* = 0.606), and slightly higher than Gemma-3–27B, although this difference did not reach statistical significance (*p* = 0.191). Llama-3.1–8B and Gemma-2–9B demonstrated comparable performance (*p* = 0.623), with Llama-3.1–8B achieving accuracy scores between 65% (60 of 92; 95% CI, 55%−74%) to 70% (64 of 92; 95% CI 60%−78%). Meanwhile, Gemma-2–2B, the smallest model evaluated, exhibited the lowest performance, with accuracy scores below 44% across all prompting strategies. These scores were significantly lower than those of Llama-3.2–3B (*p* < 0.01) and Phi-3.5-mini (*p* < 0.01), the second and third smallest models, respectively. Additionally, the smaller-scale models, i.e. those with less than 10 billion parameters, showed an apparent drop in performance when using CoT prompting and RAG, whereas the three largest models tended to improve with CoT and, to a lesser extent, with RAG. However, these differences in performance were not statistically significant for any model (*p* > 0.05). In the case of Phi-3.5-mini, an apparent effect was observed, but pairwise comparison showed it was not significant (*p* > 0.05).

**Figure 2.**
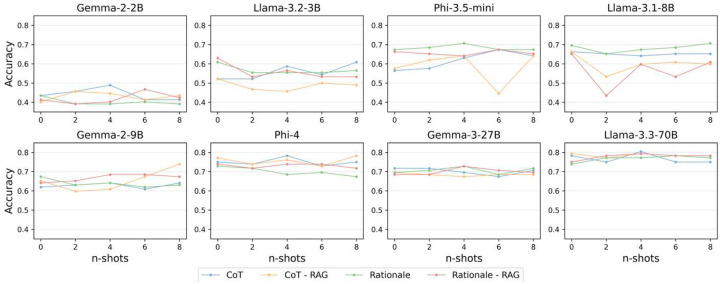
Few-shot results. Few-shot prompting accuracy scores across prompting strategies for the eight evaluated models.

Figure 2 shows the overall accuracy of each evaluated model using few-shot prompting. This experiment assessed in-context learning capabilities for rationale and CoT prompting, with and without the use of RAG, across 1 to 8 few-shot examples. Gemma-2–9B exhibited improved performance when 5 or more examples were included for CoT prompting with RAG. However, overall, there is no consistent or significant gain in accuracy associated with the included few-shot examples (*p* > 0.05). Performance variability was significant among smaller models, such as Phi-3.5-mini and Llama-3.1–8B (*p* < 0.01), but no in-context learning benefits were observed. In contrast, larger models like Gemma-3–27B and Llama-3.3-70B demonstrated a more stable accuracy across few-shot settings (*p* > 0.05) but showed no clear trend of improvement. These results suggest that the assessed LLMs do not substantially benefit from in-context learning for a highly specialized task like pediatric critical care MCQ.

**Figure 3.**
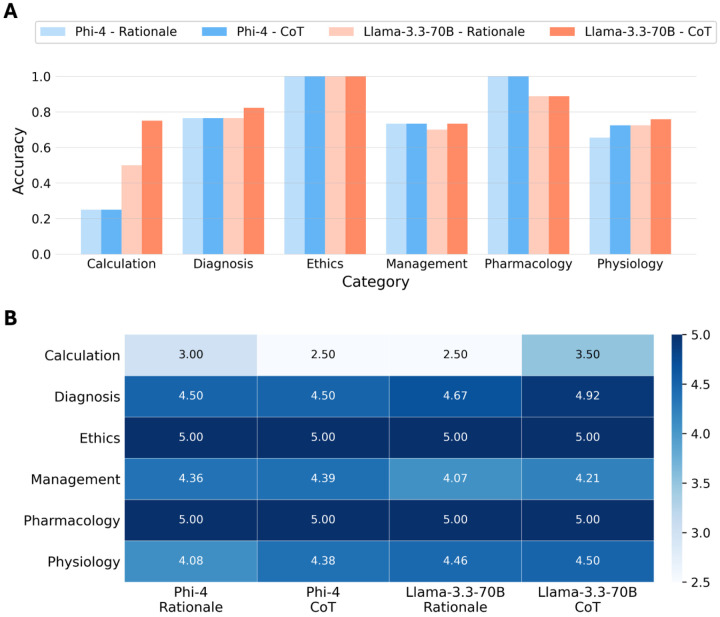
Phi-4 and Llama-3.3-70B comparison. Phi-4 and Llama-3.3-70B category-specific results for zero-shot prompting with rationale and chain-of-thought prompting, without RAG. a) Performance per dataset category. b) Average clinical reasoning quality scores per dataset category.

Based on the results across prompting strategies, Phi-4 and Llama-3.3-70B consistently outperformed the other LLMs. Therefore, subsequent analyses focused exclusively on these two models using zero-shot prompting combined with rationale and CoT prompting, without RAG. As depicted in Figure 3a, Phi-4 and Llama-3.3-70B achieved comparable performance across all dataset categories. An apparent difference was only observed in the calculation category, where Llama-3.3-70B outperformed Phi-4. Additionally, Llama-3.3-70B demonstrated a slight improvement in accuracy for diagnosis, management, and physiology with CoT prompting compared to rationale prompting. This improvement was even more noticeable for the calculation category. However, these differences were not statistically significant (*p* > 0.05). Both models achieved perfect accuracy in the ethics category.

Figure 3b shows the average clinical reasoning score, based on the predefined Likert scale, for Phi-4 and Llama-3.3-70B across categories. These scores were assigned by two pediatric specialists for a stratified subsample of 40 questions for the zero-shot prompting with rationale and CoT prompting, without RAG. Inter-rater agreement was excellent, with a Cohen’s Kappa of 0.92. The ethics category achieved a perfect score of 5.0 across models and prompting strategies, consistent with the perfect accuracy observed in Figure 3a. Overall, accuracy and reasoning scores aligned well across categories. In the calculation category, Llama-3.3-70B achieved higher accuracy, yet both models received comparable reasoning scores with rationale prompting. In contrast, for pharmacology, Llama3.3–70B received perfect clinical reasoning scores despite slightly lower accuracy. This small difference reflects that accuracy was computed on the full test set (n=92), while reasoning scores were limited to the subsample. CoT prompting yielded apparent better clinical reasoning, with average scores (SD) of 4.49 (1.01) and 4.40 (1.02) for Llama-3.3-70B and Phi-4, respectively, compared to 4.34 (1.16) and 4.33 (1.25) under rationale prompting. However, these differences were not statistically significant (*p* > 0.05).

Finally, two pediatric critical care fellows achieved overall accuracies of 73.9% and 81.5%, with an average accuracy of 77.7% and a moderate inter-subject agreement with a Cohen’s Kappa of 0.62 (Table 3). When compared to the results obtained for Llama-3.3-70B and Phi-4 using zero-shot, rationale prompting, no significant differences were observed (*p* > 0.05). Therefore, the top open-source models can achieve fellow-level performance in pediatric critical care board-style questions.

## DISCUSSION

In this study, we conducted a comprehensive evaluation of eight open-source LLMs on answering MCQs in pediatric critical care using accuracy and clinical reasoning quality scores. Our findings provide valuable insights into the potential and limitations of commonly used LLMs in this highly specialized and time-sensitive medical area that would benefit from reliable decision-support and educational tools but remains understudied.

Our results for the zero-shot prompting showed a positive correlation between model size and performance. That is, in general, larger models like Gemma-3–27B and Llama-3.3-70B achieved significantly higher accuracy scores than the smaller assessed models. Previous studies have demonstrated this tendency in both general-domain tasks and medical question-answering for MedQA, MedMCQA, and PubMedQA [28, 29]. This is reasonable as these models not only have more parameters, but their training data size is substantially larger, and therefore, they possess more foundational knowledge. However, the Phi-4 model, which is a relatively smaller model (14.7B), exhibits performance comparable to Llama-3.3-70B, and achieves marginally better accuracy than the larger Gemma-3–27B. This remarkable performance in comparison to bigger models reinforces previous findings by Abdin et al. showing Phi-4 strong reasoning capabilities, particularly in mathematical tasks [19]. This can be accredited to the model’s architecture and the training strategy, which leveraged synthetic and high-quality data along with a post-training alignment process [19]. These results suggest that besides model size, the quality and relevance of training data, and the model design and architectural choices are critical for highly specialized domains. There might be a better representation of pediatric critical care knowledge in the pre-training corpus of Phi-4, even if it is significantly smaller than the pre-training corpus of Llama-3.3-70B and Gemma-3–27B.

Regarding the different prompting strategies in the zero-shot setting, our evaluation showed that CoT prompting yielded a marginal improvement in performance of larger models but had a slight negative impact on smaller models (<10B) including Gemma-2–2B, Llama-3.2–3B, Phi-3.5-mini, Llama-3.1–8B, and Gemma-2–9B. This aligns with prior work by Wei et al. and Kojima et al., who used multiple general-domain benchmark reasoning tasks to demonstrate that CoT prompting enhances reasoning capabilities as model size increases [22, 25]. In other words, models must be sufficiently large to effectively leverage the step-by-step reasoning process that CoT encourages. Notably, Phi-4 was the only relatively smaller model to marginally benefit from CoT prompting likely due to model’s architecture, training data, and training strategies used for its development. These findings show that smaller models may require domain-specific fine-tuning to benefit from this prompting strategy [19].

Similarly, RAG did not produce consistent performance improvements for smaller models and, in some cases, led to a slight performance degradation. This suggests that introducing external information may overwhelm the reasoning capabilities of smaller models, resulting in confusion rather than improved performance. In contrast, larger models demonstrated marginal gains, indicating a better ability to integrate retrieved content. Interestingly, Phi-4 again deviated from this pattern, showing marginal improvements with RAG. Therefore, a more tailored and sophisticated retrieval mechanism, like the medical graph-based RAG proposed by Junde Wu et al., along with more comprehensive PICU resources, might be necessary to incorporate relevant contextual information that benefits the LLMs for pediatric critical care MCQ answering [30].

In-context learning, evaluated via few-shot learning across different prompting strategies, was largely ineffective for this task. Even though an improvement in performance is typically expected in general domains and more general medicine tasks [21, 31–34], for the highly specialized and complex nature of pediatric critical care, few-shot learning might be insufficient to effectively enhance model performance. Additionally, the nuanced age-related physiological differences found in pediatric patients might limit the in-context learning capabilities. Our findings suggest that relevant domain-specific, high-quality PICU knowledge is required in the pre-training corpus of the models to improve their performance.

Our results for the category-specific assessment for Phi-4 and Llama-3.3-70B with zero-shot prompting with rationale and CoT prompting, without RAG, demonstrated strong alignment between accuracy and clinical reasoning capabilities based on the Likert scale. This is particularly important for real-world clinical applications in pediatric critical care since the models are selecting the correct answer through correct, relevant clinical reasoning. Furthermore, both models achieved comparable performance for both evaluation metrics despite the significant difference in their sizes, with a notable exception in the calculation category, where both achieved lower results, but Llama-3.3.−70B outperformed Phi-4. For this category, both models struggled applying the appropriate formula and selecting the correct values to input. Similar difficulties in mathematical reasoning within the context of medical calculations were identified by Khandekar et al [35]. Conversely, both models had a perfect performance in the ethics category, which suggests that medical ethics knowledge is likely well represented in the pre-training data and can be successfully applied to the pediatric critical care domain. Importantly, for several questions with lower clinical reasoning scores, the models used a generally correct principle that was inappropriate for the described patient, who actually required a different management strategy due to their distinct physiology. These findings further underscore the need to incorporate domain-specific data into the pre-training corpus to improve model performance in pediatric critical care.

Finally, Llama-3.3.−70B and Phi-4 achieved accuracies not significantly different to two pediatric critical care fellows when using zero-shot learning with rationale prompting strategy. These results constitute an important benchmark for the potential utility of open-source models for well-defined tasks and warrant further investigation as potential decision-support tools and complementary educational resources for pediatric critical care training programs.

Our work does have some limitations:

1. Our 100 MCQ dataset, while carefully created by pediatric specialists, is relatively small. A larger dataset could be a better and more comprehensive representation of pediatric critical care knowledge, and thus, benefit the assessment and strengthen our findings.
2. Our evaluation focused exclusively on MCQs, which might not accurately reflect the complexity of real-world PICU clinical reasoning and decision-making.
3. The clinical reasoning quality evaluation was conducted by two pediatric specialists. Similarly, the MCQ completion for human comparison was performed by two pediatric critical care fellows. Having a larger panel of experts across different training levels and a more extensive evaluation criteria could benefit the robustness and reliability of our assessment in this context.
4. We focused on open-source models below 70B parameters, excluding widely used larger proprietary models like GPT-4, Claude 3.7 Sonnet [36], and PaLM 2, which might have a different performance for this task. However, our focus on the evaluated open-source models was deliberate, given their potential for real-world clinical applications where privacy and transparency are critical concerns [37, 38].

Despite these limitations, we demonstrated that smaller models like Phi-4 are promising for the development pediatric critical care decision-support tools in different settings, including resource-constrained environments. Importantly, prior to implementation in a clinical support role, these models must have rigorous patient specific testing to ensure model output is accurate across use cases.

Future research should explore fine-tuning smaller models like Phi-4 using a high-quality, PICU-specific corpus. There is a need for more sophisticated and comprehensive evaluation frameworks that includes a variety of relevant real-world PICU scenarios and clinical simulations to validate the utility of these models as clinical decision-support tools and educational resources for trainees in pediatric critical care. Additionally, any future deployment in clinical settings require human oversight and institutional safeguards to ensure safe and effective use in pediatric patient care.

## CONCLUSION

We evaluated eight open-source LLMs for pediatric critical care MCQ answering and found notable differences in accuracy and clinical reasoning. The larger models, Llama-3.3-70B and Gemma-3–27B, as well as the smaller Phi-4 model, showed promising potential as decision-support tools and educational resources in the PICU. However, foundational knowledge gaps remain and must be addressed before adaptation as educational tools and clinical integration.

## Supplementary Material

Supplementary Files

This is a list of supplementary files associated with this preprint. Click to download.

- llmpicusupplement.docx

## Figures and Tables

**Table 1.** Large Language Models description.

Model	Parameters, B	Context Length – Tokens, K	Training Data Size – Tokens, T
Gemma-2–2B	2.61	8	2
Llama-3.2–3B	3.21	128	9
Phi-3.5-mini	3.82	128	3.4
Llama-3.1–8B	8.03	128	15
Gemma-2–9B	9.24	8	8
Phi-4	14.7	16	9.8
Gemma-3–27B	27.4	128	14
Llama-3.3-70B	70.6	128	15

Characteristics of the eight evaluated LLMs. B denotes billions, K thousands, and T trillions.

**Table 2.** Zero-shot accuracy results.

Model	Rationale	Rationale RAG	Chain-of-Thought	Chain-of-Thought RAG	P-value
Gemma-2–2B	43.48	41.30	43.48	40.22	0.908
Llama-3.2–3B	60.87	63.04	52.17	52.17	0.091
Phi-3.5-mini	67.39	66.30	56.52	57.61	0.037
Llama-3.1–8B	69.57	65.22	66.30	66.30	0.717
Gemma-2–9B	67.39	64.13	61.96	65.22	0.630
Phi-4	72.83	73.91	75.00	77.17	0.677
Gemma-3–27B	68.48	69.57	69.57	71.74	0.843
Llama-3.3-70B	**73.91**	**75.00**	**78.26**	**79.35**	0.303

Zero-shot prompting overall accuracy for rationale and chain-of-thought prompting, with and without RAG, for the eight studied LLMs. The best performance for each setting is bolded, and the second-best is underlined.

**Table 3.** Category-specific accuracy results.

Subject	Calculation	Diagnosis	Ethics	Management	Pharmacology	Physiology
Fellow 1	75.00	70.59	100.00	76.67	88.89	65.52
Fellow 2	50.00	88.23	66.67	86.67	77.78	79.31
Phi-4	25.00	76.47	100.00	73.33	100	65.51
Llama-3.3-70B	50.00	76.47	100.00	70.00	88.89	72.41

Category-specific accuracy comparison for two pediatric critical care fellows and the two best-performing LLMs.

## Data Availability

The datasets used and/or analyzed during the current study are available from the corresponding author on reasonable request.

## List of Abbreviations:

CoT: Chain-of-Thought
EHR: Electronic Health Record
LLM: Large Language Model
MCQ: Multiple-Choice Question
PICU: Pediatric Intensive Care Unit
RAG: Retrieval Augmented Generation
